# Minutes of the Third Regular Meeting of the Western Dental Society

**Published:** 1857-06

**Authors:** 


### Proceedings of Societies.

### MINUTES OF THE THIRD REGULAR MEETING OF THE WESTERN DENTAL SOCIETY.

St. Louis, May 20, 1857.

The Society met as per adjournment, in the Temperance Hall, at 10½ o'clock, and was called to order by the President. An abstract of the minutes of the last meeting, held at Chicago, was read by the Secretary.

The Treasurer made the following report, as the state of his account, and requests the auditing committee to examine the same:

| | |
|---|---|
| Received for membership, | $185,00 |
| The disbursements for various purposes amount to | $126,06 |
| Leaving a balance in the Treasury of | $58,94 |

All of which is respectfully submitted,

A. Blake, Treas.

Which on motion was received.

The accounts of the Treasurer not having been audited, the above motion was reconsidered, and the account referred.

On motion of Dr. Allport, Dr. Taft was elected an honorary member of this society.

Dr. Allport moved that a business committee of three be appointed, which was carried, and Drs. Allport, Spalding and Peebles were appointed said committee.

On Motion of Dr. Spalding, a recess was taken to give the committee time to report.

After the time had expired, the meeting was called to order, and the committee reported business for the action of the Society, as follows:

### REPORT OF BUSINESS COMMITTEE.

I. Election of members.

II. Election of officers for the ensuing year.

III. Miscellaneous business.

1st. The best method of arresting decay in the teeth.

2d. Causes and treatment of diseased gums.

3d. Cheoplasty.

4th. Difficult Dentition.

5th. In what cases, if any, is it answerable to preserve the fangs of the natural teeth for the reception of artificial crowns.

The committee reported the following gentlemen as having passed satisfactory examinations, and recommended them for membership : Dr. Hubbard, of Quincy, Ill., Drs. A. M. Leslie, C. B. Porter, D. L. Levett, of St. Louis, who were unanimously elected.

On motion it was decided to proceed to the election of officers in the usual way.

Pending the election, it was, on motion, ordered that a committee of two be appointed to invite Dentists residing in the city, and who are not members, and other members of the profession who might be in the city, to attend upon the deliberations of the society. Drs. Silvers and Leslie were appointed a committee.

On motion of Dr. Spalding, it was resolved that the officers be elected by an informal ballot, which resulted as follows:

*President*—DR. ISAIAH FORBES.

*First Vice-President*—DR. H. N. LEWIS.

*Second do* DR. C. B. PORTER.

*Recording Secretary*—DR. H. E. PEEBLES.

*Corresponding Secretary*—Dr. H. J. B. MCKELLOPS.

*Treasurer*—Dr. A. BLAKE.

*Executive Committee*—DRS. H. BARRON, W. W. ALLPORT, S. DUNHAM, E. HALE, Sen., and —— BULL.

Dr. Hale, the former President, conducted the President elect to the chair, who delivered a very appropriate address for the occasion.

On motion of Dr. Perrine, the thanks of this Society were tendered Dr. Hale, for the ability with which he has conducted the proceedings of the Society for the last year.

Dr. Allport moved that the chair appoint five delegates to the American Dental Convention, to meet in Boston in August next.

The Society adjourned to meet at 3 o'clock, P. M.

AFTERNOON SESSION.

In pursuance of adjournment, the Society met at three o'clock, P. M.

President in the chair. The minutes of the morning session were read and adopted.

Reports of Committees being called for, the Committee on By-Laws reported as follows:

BY-LAWS.

ARTICLE 1. The constitution of the W. D. Society shall be deemed the fundamental law of this Society.

ARTICLE 2. This Society shall be governed by parliamentary usage.

ORDER OF BUSINESS.

ARTICLE 3. 1st. The minutes of the preceding meeting shall be read.

2d. Reports from Treasurer and standing committees.

3d. Election of members.

4th. Election of officers.

5th. Unfinished business.

6th. Miscellaneous business.

7th Reading essays and papers.

8th. Payment of dues.

9th. President's address.

10th. Reading, correcting and adopting the minutes.

ART. 4 No member shall speak more than once on the same subject, till all have had an opportunity to speak.

All of which is respectfully submitted.

W. W. ALLPORT,

H. E. PEEBLES,

I. FORBES.

} Committee.

On motion of Dr. Spalding, the report was accepted. The By-Laws were then read, amended and adopted article by article.

Miscellaneous business being then in order, Dr. Perrine offered a preamble and resolution on local societies.

WHEREAS, it is evident to the observing Dentist that our profession has been more rapidly advancing in usefulness and knowledge since the organization of the American Dental Society than at any former period of time. And, whereas, this fact is mainly attributable to organized association and its concomitants, the general and firm interchange of sentiment and diffusion of knowledge, through free discussion, the school and the press. And, whereas, the multiplication of Dental Societies in our own country has greatly increased the facilities for acquiring correct information, and emulating individual enterprise. Therefore,

*Resolved*, That we approbate, encourage and advise the formation of local Societies in all places where they can be sustained.

The motion was seconded by Dr. Allport, and adopted.

Dr. Allport moved that a committee of five be appointed, with Dr. Taft as its chairman, to examine, test and report on "Cheoplasty." Adopted.

The President appointed Drs. Taft, Allport, Lewis, McKellops, Leslie, and Dunham as said Committee.

Dr. Perrine moved that a committee of three be appointed to consider and report the subject of patenting by the Dental profession.

Drs. Perrine, Barron and Hubbard were appointed. No more miscellaneous business being presented, the regular order of business was taken up.

The best manner of arresting decay in teeth was then discussed.

On motion of Dr. Barron, the Secretary called the roll, and each member present responded, and gave his views upon some special case of filling, filing or cutting, and polishing, aided by diagrams on blackboard.

Adjourned to meet at 8 o'clock, P. M., in room of St. Louis Dental Society ; and also to meet in this Hall at 9 A. M., to morrow.

EVENING SESSION.

Pursuant to adjournment, the Society met in the rooms of the St. Louis Dental Society, at 8 P. M. Second Vice-President, Porter, in the chair.

Discussion on tooth filling continued, which took rather a wide range, embracing collateral issues.

Dr. Perrine presented the name of Dr. I. B. Branch, of Galena, Ill., for membership.

Adjourned to meet at the Hall at 9 A. M. to-morrow morning.

SECOND DAY—MORNING SESSION.

Pursuant to adjournment, the society met in their Hall, at 9 o'clock, A. M. President in the chair.

The minutes of the afternoon and evening sessions were read and adopted.

Dr. Geo. H. Silvers presented the name of J. W. Fulton, of Paris, Ill ; Dr. E. Hale, Jr., presented the name of Dr. McCuen, of Louisiana, Mo., and Dr. Perrine presented the names of Dr. A. Blakesly, of Utica, N. Y., and Dr. C. M. Forbes, as candidates for membership.

MISCELLANEOUS BUSINESS.

Dr. Perrine offered the following resolution :

*Resolved,* That all members present having performed any operation requiring peculiar manipulation, instruments or any thing new, may have the opportunity to present them after the order of business.

After which Dr. I. B. Branch, of Galena, Ill., was elected an active member, and Dr. A. Blakesly, of Utica, N. Y., an honorary member.

Dr. Spalding received a letter from Dr. A. W. French, of Springfield, Ill.

On motion of Dr. Perrine, the letter was read, and the following resolution adopted.

*Resolved*, That the exhibition of specimen cases of dental mechanism at Cattle Shows, Mechanics' Fairs, and other similar places, can but be regarded as derogatory to our profession, and will never be resorted to by any intelligent Dentist who desires its elevation.

After which the discussion on the cause and treatment of diseased gums was resumed. The roll was called, and each member responded, making the discussion interesting, and somewhat amusing.

On motion of Dr. Allport, the discussion of this subject was discontinued after the remarks of Dr. E. Hale, Sen.

The executive committee presented the name of C. M. Forbes for membership, who on ballot was elected.

On motion of Dr. Spalding, a committee of five was appointed on dental fees, to report to-morrow. Drs. Spalding, Allport, Lewis, Branch and Porter were appointed said commmittee.

On motion of Dr. Allport, the hours from 3 to 5 o'clock this afternoon, were appropriated to speaking on any or all subjects proper to come up.

Adjourned to meet at 3 o'clock, P. M.

SECOND DAY—AFTERNOON SESSION.

Met agreeable to adjournment, at 3, P. M.

1st Vice President in the chair. On motion, the reading of the minutes of the morning session was dispensed with.

On motion of Dr. Dunham, the members were limited to ten minutes.

After this discussion, the society proceeded to the consideration of instruments, apparatus, &c.

Dr. I. Forbes, exhibited an instrument for drying out partially filled cavities. Dr. E. Hale, jr., presented an instrument used with advantage in swaging plates. Dr. Perrine exhibited a flask for holding a case of teeth while soldering, and, also, a book for recording dental operations. Dr. Peebles, presented an instrument for holding a polishing stone in finishing fillings.

The time having expired for miscellaneous discussions, on motion, it was extended half an hour. And when this had expired the discussion was further extended.

Adjourned till 9 o'clock, A. M. to-morrow.

THIRD DAY—MORNING SESSION, FRIDAY, 22.

Meeting called to order, pursuant to adjournment, by the 2d Vice President, Dr. Porter.

The minutes of the previous session were read. Miscellaneous business was then taken up. Dr. Perrine offered a preamble and resolutions on the death of the late Dr. Hullihen.

WHEREAS, By the inscrutable decrees of Divine Providence, there has been taken from his career of usefulness on earth, to that bourne from whence no traveler returns, Dr. Hullihen, of Wheeling, who by his superior skill, and attainments, had become a beacon light in his profession, who by his usefulness and generous manifestation, had not only endeared himself to those whom his skill had benefitted, but to every member of his profession, and which will be imbedded in their grateful hearts, until time shall be no more. Had become a radiant star in the bright galaxy of surgical dentistry, ever maintaining that dignity in his profession which enabled him to compel others of a kindred profession to submit to his performing every operation that rightly belonged to dental surgery. Therefore be it,

*Resolved*, That the Western Dental Society, deeply sympathize with the relations of our brother, so suddenly taken in the bloom of life.

*Resolved*, That a copy of these resolutions be transmitted through the Secretary, to the relatives of the deceased.

*Resolved*, That these resolutions be published in all the Dental publications in the Union.

The name of Dr. Hannaman, of Shelbyville, Ill., was presented by Dr. Sturges for membership, and was referred to the Executive Committee.

The subject of "Difficult Dentition," was called up, but no discussion elicited.

Reports of special committees were called for, and the committee on dental patents reported, which was received, and after some discussion, part of it was laid on the table, and the balance referred back to said committee.

The committee on "Cheoplasty" reported, which was received and adopted.

The committee to whom was referred for consideration the style and construction of artificial dentures, denominated "*Cheoplastic*," would respectfully submit the following report:

We have examined the subject as thoroughly as time and opportunity would permit.

We are unadvised in regard to the composition of the material of the base. It seems sufficiently dense, and it is stiff enough when formed into thin plates for all practical purposes.

It is not more readily bent or changed in form, than a swaged gold plate. It possesses in our opinion, when properly constructed, quite as much, if not more strength, than a piece of work well constructed of gold.

We do not entertain a doubt about the durability of the work, so far as strength is concerned.

In regard to the indestructibility of the metal, we have not much to say; because we do not know much about that. We are acquainted with the fact, however, that it has been in the mouths of various persons for several months, without any perceptible change. And, it is worthy of notice, that some of those persons are competent to detect even the slightest change, either in the appearance of the metal, after it has been worn in the mouth, or by any thing peculiar, while in the mouth.

From what knowledge we have obtained in regard to this metal, we concur in the opinion, that it may be used in the majority of cases with impunity, and, perhaps, in all, so far as destructibility is concerned.

There are two or three particulars, in which it possesses excellencies not to be found in other styles of work. There, are no interstices or points for lodgment of food or other substances. In the construction of the pieces, the base is adapted to the teeth and not the teeth to the base; and that adaptation is perfect. Consequently, it is easily kept clean. Indeed, the same care required to keep the natural teeth clean, would preserve this in perfect condition. We consider the adaptation to the gums more perfect and more easily obtained, than by any other method. The metal neither contracts nor expands, when passed from a low to a high temperature. It is cast to the model, and runs very sharp, so that it makes a perfect counterpart of the model. Hence, a perfect fit is the result, if an accurate impression of the mouth has been taken. Any desired shape can be given to it, for the more perfect restoration of the features. Any kind of teeth may be used in its construction. Those teeth in sections or blocks, seem to be well adapted to it, and the ordinary gum or plain teeth may be used with facility; but teeth are now manufactured especially for this kind of work, so formed that it is impossible after a piece is constructed to remove them from the plate, without either cutting away, or melting the plate, or breaking the teeth.

The facility with which it may be constructed, is particularly worthy of notice; though we doubt not under the excitement of unanticipated success, impressions have been made on the minds of many on this point, that will not be completely verified in the execution of the work. And we would suggest to those interested in the matter, that they should not make this point *too* prominent, though in *their* particular cases, all they affirm may be correct. Yet, we hesitate not, to say, from our present knowledge of the work that artificial dentures can be constructed by this mode, with greater facility than by any other efficient mode in use. A tooth can be replaced, or any injury of the plate repaired without difficulty, and in a short time.

It has been objected that the work is too heavy; but it is not more objectionable in this respect than some other kinds, for instance, continuous gum work, or blocks on heavy gold plate.

There are some patients that will complain of any denture, however light, in the mouth. With such, it is the *presence* of anything in the mouth, and not the weight that causes the objection. A heavy plate and perfect fit is far preferable to a light plate and an imperfect fit. It is also objected, that the metal is not sufficiently indestructible. On this point we have nothing to say. Our experience in regard to this being very limited. On this particular, we rely mainly on those who have more experience, and who have, for a much longer period and under more favorable opportunity, directed their attention in this channel.

All of which is respectfully submitted,

J. Taft,<br>

H. N. Lewis,<br>

S. Dunham, } Committee.<br>

A. M. Leslie,<br>

H. McKellops,

The auditing committee on Treasurer's books made the following report, which was adopted:

"The committee authorized to examine the Treasurer's report have attended to that duty, and find his account correct."

H. McKellops, } Committee<br>

S. Dunham,

The committee on Dental Patents, reported, which was received and adopted.

Whereas, The multiplication of Dental Patents in our country, seems to be a source of reasonable apprehension, lest the honor and standard of our profession be lowered. Therefore,

*Resolved*, 1st. That the principles of patents as usual in our profession of late, is unprofessional, and not consistent with an honorable profession.

*Resolved*, 2d. That we do not consider it wrong for any member of our profession, who may invent a useful article in the manufacture of artificial structures or instruments, to patent the same, provided he confine his patent to the sale of the material, at a reasonable charge, and not to the use of the same.

Dr. Mc Kellops, offered the following resolution:

WHEREAS, In consequence of great benefit enjoyed by the dental profession, from the peculiar manipulation of Dr. Arthur in filling teeth, we feel it a duty and privilege to manifest our approbation of these benefits, in some tangible form. Therefore,

*Resolved*, That a committee be appointed to prepare a testimonial of thanks, which shall be presented to Dr. Arthur, by the Western Dental Society. Adopted.

Committee, Drs. McKellops, Taft and Allport, and on motion the President was added to that committee.

Dr. Peebles offered the following:—

*Resolved*, That a committee of three be appointed to present at an early period, the regular order of business, for the next annual meeting. Adopted.

DR. PEEBLES,<br>DR. PERRINE, } Committee.<br>DR. BLAKE.

The executive committee examined and presented Dr. G. Hannaman for membership, who upon ballot was declared duly elected.

The committee on Fees and Professional etiquette reported.

REPORT OF COMMITTEE ON FEES AND PROFESSIONAL ETIQUETTE:

*Resolved*, That the long experience and thorough course of study needful to render the dental practitioner competent to discharge the responsible duties of his profession, entitle him to a fair compensation for services rendered. That cheap dental operations, either surgical or mechanical are dear and usually unsatisfactory to the patient, and tend to degrade the profession, in the estimation of the community.

*Resolved*, That this society recommend the formation of local societies, for the purpose of regulating fees, and at the same time promoting harmony and good feeling among the members of the profession.

*Resolved*, That a dentist offering to travel out of his regular field of practice to perform operations in the field of a brother practitioner for less than the usual fee is discourteous and unprofessional. C. W. SPALDING.

The above report was adopted.

The President appointed Drs. Taft, Allport, Spalding, Branch, McKellops and Forbes, as delegates to the American Dental Convention.

On motion of Dr. Perrine, it was

*Resolved*, That when we adjourn, we do so to meet at Quincy, Ill., on the third Tuesday in July, 1858.

Adjourned to meet at 2½ P. M.

AFTERNOON SESSION, 2½ P. M.

Pursuant to adjournment, the meeting was called to order, President in the chair.

The minutes of the morning session were read and adopted.

The subject of "Difficult Dentition," was again called up, and elicited a very interesting discussion, and a pledge from the members to aid in the acquisition of knowledge on this subject, by experiment during the ensuing year.

On motion of Dr. E. Hale, jr., the subject of difficult dentition is made the special order of business for the next meeting.

The report of the committee, on Prof. Arthur's plan of filling teeth was presented and adopted.

*Resolved*, That to Dr. Arthur and he alone, the dental profession is under obligation for his liberality in laying before the profession the principle of using and welding together annealed gold, by the use of serrated pointed instruments, and that this society desire to express their thanks to him for this one, of the real improvements in the mode of operating.

H. McKellops.
J. Taft.
W. W. Allport.

On motion of Dr. Allport, we now occupy the remainder of this session in free and promiscuous discussion, no member occupying more than ten minutes in one speech.

On motion of Dr. Allport. A committee of five was appointed to test local anæsthesia, and to report at the next meeting. Allport, McKellops, Dunham, Ferris and Porter, were appointed on that committee.

The minutes were read, corrected and adopted.

On motion, adjourned to meet in Quincy, Ill. July 22d, 1858. ISAIAH FORBES, President.

H. E. Peebles, Secretary.

P. S. After the meeting adjourned, a lot of specimen teeth and tin work, also a platinum case, also an old broken tin case, which had been used for a number of years, was received from Dr. W. G. Oliver, of Buffalo, N. Y., and presented by Dr. Perrine, who also read a lengthy letter from the same gentleman, to the various members who were still in the city. P.

---

